# Multidisciplinary survey on use of feeding tubes in head and neck cancer patients undergoing chemoradiotherapy in Germany—the SUFEETUBE project

**DOI:** 10.1007/s00066-024-02206-w

**Published:** 2024-02-21

**Authors:** Anastassia Löser, Alexander Fabian, Alexander Rühle, Alexander Thieme, Andrea Baehr, Lukas Käsmann, Inga Zwaan, Birte Kahle, Tamer Soror, Ameya Kunte, Niloufar Seyedi, Maxim Kebenko, Christoph Seidel, Friederike Dierks, Linda Krause, Karl-Ludwig Bruchhage, Dirk Rades

**Affiliations:** 1 Department of Radiotherapy, University Medical Center Schleswig-Holstein Campus Lübeck, Ratzeburger Allee 160, 23538 Lübeck, Germany; 2 Department of Radiation Oncology, University Hospital Schleswig-Holstein/Kiel, Arnold-Heller-Straße 3, 24105 Kiel, Germany; 3 Department of Radiation Oncology, Medical Center, Faculty of Medicine, University of Freiburg, Robert-Koch-Straße 3, 79106 Freiburg, Germany; 4 Department of Radiation Oncology, University of Leipzig Medical Center, Stephanstr. 9a, 04103 Leipzig, Germany; 5 Department of Radiation Oncology, Charité-Universitätsmedizin Berlin, Augustenburger Platz 1, 13353 Berlin, Germany; 6 Department of Radiotherapy and Radiation Oncology, University Medical Center Hamburg-Eppendorf, Martinistraße 52, 20246 Hamburg, Germany; 7 Clinic and Polyclinic for Radiotherapy and Radiooncology, LMU Clinic, LMU Munich, Marchioninistraße 15, 81377 Munich, Germany; 8 Department of Stem Cell Transplantation, University Medical Center Hamburg Eppendorf, Martinistraße 52, 20251 Hamburg, Germany; 9 Department of Hematology and Oncology, University Hospital Schleswig-Holstein, Ratzeburger Allee 160, 23538 Lübeck, Germany; 10 Department of Oncology, Hematology and Bone Marrow Transplantation with Division of Pneumology, University Medical Center Hamburg-Eppendorf, Martinistraße 52, Hamburg, Germany; 11 Institute of Medical Biometry and Epidemiology, University Medical Center Hamburg-Eppendorf, Martinistraße 52, 20246 Hamburg, Germany; 12 Department of Otorhinolaryngology, Head and Neck Surgery, University Hospital Schleswig-Holstein, Ratzeburger Allee 160, 23538 Lübeck, Germany

**Keywords:** Malnutrition, Dysphagia, PEG, Gastrostomy tube, Mucositis

## Abstract

**Background and objective:**

Data on enteral tube feeding in head and neck cancer (HNC) patients undergoing chemoradiotherapy vary considerably between German institutions. This survey aims to investigate the management of feeding tubes in an interdisciplinary context across Germany.

**Materials and methods:**

Between December 2022 and May 2023, 70 participants (42 radiation oncologists, 12 medical oncologists, 14 head and neck surgeons, and 2 physicians covering several specialties) responded to our web-based survey. In addition to the type of institution (university hospital, private practice, etc.), their age, and professional experience (in years), participants were asked several questions on the indication and institutional policy for tube placement and management (prophylactic/reactive nasogastric or gastrostomy tube). All questions were mandatory single- or multiple-choice questions, while additional comments were possible by email.

**Results:**

Most participants were employed at a university hospital (*n* = 52; 74.3%) and came from a radiation oncology background (*n* = 42; 60%). Fifty-four contributors (77.1%) reported that no nutritional risk screening prior to chemoradiotherapy was routinely performed, and 71.4% (*n* = 50) stated that no standardized protocol was used at the institution to set the indication for tube placement. Generally, policies and methods of tube feeding vary considerably between the individual institutions and specialties. However, the majority (*n* = 56, 80%) recommended a prophylactic percutaneous enteral gastrostomy (PEG) tube to their patients before chemoradiotherapy. Still, there was no consistent trend regarding the approach for reactive tube feeding.

**Conclusion:**

The policies and methods of tube feeding vary considerably between the individual institutions and specialties in Germany. In the era of individualized medicine, uniform protocols are difficult to establish. However, a baseline nutritional risk screening could simplify decision-making in clinical practice.

## Introduction

Head and cancer (HNC) patients undergoing definitive or adjuvant chemoradiotherapy (CRT) with curative intent are at a high risk of malnutrition. Experience shows that the prevalence of malnutrition increases up to 88% during ongoing therapy [1]. This happens due to pre-existing tumor-associated symptoms, and/or arising CRT-associated side effects like mucositis or dysphagia. Malnutrition is known to be associated with poorer overall survival as well as lower quality of life in HNC patients [2–5].

If oral food intake can no longer be adequately guaranteed, there are two routes for compensation: while enteral nutrition involves providing nutrients via the gastrointestinal tract, either orally (e.g., by means of oral supplements) or through a tube, parenteral nutrition involves delivering nutrients through intravenous administration (e.g., via a port catheter). The choice between these methods depends on the patient’s clinical condition, the functionality of the gastrointestinal tract, and the overall nutritional requirements. Enteral feeding options include percutaneous endoscopic gastrostomy (PEG; or, in some cases percutaneous endoscopic jejunostomy, PEJ) or a nasogastric tube.

Since radiation-induced dysphagia is known to be a risk factor for malnutrition [6], it has been the subject of several clinical studies [7, 8]. Barbon et al. concluded that prophylactic movement of the pharyngeal muscles during oral ingestion as well as swallowing exercises may prevent dysphagia [8]. There are specialized validated nutritional screening tools to identify patients at risk, e.g., the Nutritional Risk Screening (NRS2002) or the Malnutrition Universal Screening Tool (MUST) [9].

Internationally, there is no consensus on the best timing and method of tube feeding for HNC patients undergoing CRT [10]. Similarly, there is no uniform consensus throughout Germany. In their systematic review, McClelland et al. investigated the optimal timing of feeding tube placement [11]: in some oncology centers, treatment preparation before planned CRT requires insertion of a (prophylactic) PEG tube in addition to a port catheter, while others favor initiation of tube feeding when the patient develops significant weight loss or dysphagia (reactive) [11]. The question of the correct timing of a PEG tube and its influence on the patients’ quality of life including patient-reported outcomes was investigated in the so-called Swall PEG study, a phase III trial. The results have not yet been published [12].

After reviewing 26 manuscripts, Bossola et al. concluded that the use of nasogastric (NGT) and PEG tubes was comparable regarding nutritional and oncologic endpoints [13]. On the one hand, PEG tube placement is known for its potential minor (e.g., peristomal leaks, infection) or major complications (e.g., bowel perforation, gastrointestinal hemorrhage, or even procedure-related mortality) [14]. On the other hand, NGT dislocate more frequently, cause more patient discomfort, and promote aspiration pneumonia [13, 15].

To identify current policies of enteral nutrition (EN), (mostly national) surveys have been launched in the past. Probably the largest survey of radiation oncologists’ attitudes toward PEG tubes comes from China, where 361 radiation oncologists from 26 hospitals participated [16]. However, there are also some European representatives addressing this issue: Dragan et al. distributed their survey to 24 Belgian radiation oncology departments, observing a disparity among treatment centers [10]. In 2021, Ilmarinen et al. published the results from their survey on current policies and perceptions of EN in HNC patients under CRT in Nordic countries. In total, they analyzed 42 responses (21 oncologists and 21 head and neck surgeons each) [17]. The authors reported disparity in the practices for tube feeding in HNC management among the Nordic countries. They recommended that unified protocols for tube feeding should be established for this patient population [17]. One year later, Bozzetti et al. announced their results originating from a 19-item multinational survey with more than 10 contributing European (and non-European) countries. It is worth mentioning that Germany contributed less than 1% and therefore was not mentioned separately [18].

Especially in Europe, data on physicians’ choice of feeding tubes in HNC treated with CRT are very limited. To the best of our knowledge, there is no comparable study on the current policies in tube feeding in Germany. The aim of this nationwide interdisciplinary survey was to gain knowledge on physicians’ attitudes concerning enteral feeding tubes at different German institutions.

## Methods

### Survey design

Between December 2022 and May 2023, we conducted a web-based survey consisting of 20 questions (16 multiple-choice questions, 2 Net Promoter Score [NPS]® (Satmetrix Systems, Inc., Bain & Company, Munich, Germany) questions, and 2 open-ended questions) using Microsoft Forms (Microsoft Corporation 2023, Redmond, WA, USA) addressing current policies and routes of EN in HNC patients undergoing CRT.

During recruitment, no exclusion of certain institutions was performed. Therefore, participants were based not only at university hospitals, but also at other (non-university municipal) hospitals, medical care centers, and physicians’ private practices. Requests for participation were sent by email with a cover letter explaining the background of the survey. Radiation oncologists, head and neck surgeons, or otolaryngologists (ear, nose, throat [ENT] doctors) as well as medical oncologists working in Germany were eligible to participate.

Each of the coauthors recruited at least five additional colleagues from the departments of oncology, radiation oncology, and ENT. Answering the survey was possible via a link or QR code. Participants were asked to forward the survey link to other colleagues in the said specialties. Responses to the survey were captured anonymously. However, on a voluntary basis, participants were able to leave their names.

The survey was subdivided into three sections: the first section consisted of six general questions on the participant’s age, gender, clinical experience (in years), specialty, the number of annually treated HNC patients, and the type of institution. The second section included seven questions on current policies of tube feeding and nutritional pretreatment screening. The last section involved another seven questions asking for the respondent’s personal expert opinion involving three patient cases. Questions no. 13, no. 16, and no. 17 were included analogous to the publication by Ilmarinen et al. [17]. As the data available on this topic are limited, we wanted to create comparability with previously published literature.

Patient case no. 20 comes from the authors’ everyday clinical practice. As many possibilities can certainly arise here, in our opinion, this question reflects the complexity and controversy of the topic of enteral feeding tubes. Except for two open questions, comments could be made via email to the corresponding author.

### Sample size calculation

Prior to conducting the survey, we performed a sample-size calculation by means of the finite population correction factor or FPC formula:

$$n=(Z^{2}*p*(1-p))/E^{2}{,}$$

where *n* corresponds to the sample size, Z is the Z‑value for the desired confidence level (for our survey: 1.96 for a 95% confidence level), *p* is the estimated proportion of doctors answering the SUFFETUBE survey completely, and E the desired confidence interval or margin of error.

We assumed a poor response rate, namely that only 50% of physicians would answer our survey completely (*p* = 0.5). To define the population size, it was assumed that there are 6557 working ENT physicians (as of 2022 [19]), 1564 working radiation therapists (as of 2022 [20]), and 2758 practicing medical oncologists (as of 2020, corresponding focus for hematology and oncology, [21]) in Germany. Therefore, the resulting population size comprised 10,879 practicing physicians of three different medical specialties. We assumed a confidence interval of 12%, which resulted in a sample size of 66.7 needed participants after entering all values into the upper formula.

### Further statistical analyses

Data were analyzed using descriptive statistics presenting them as means with standard deviation (SD) or percentages where appropriate. Bar charts were generated using Excel (version 2304, Microsoft Corporation 2023, Redmond, WA, USA).

The distribution of a nominally scaled sample was calculated using a test for binomial distribution. In the case of multiple expression levels, the chi-square test was applied. The Mann–Whitney U test was used to compare nonparametric samples. To decide whether a statistically significant association existed between two categorical variables, Fisher’s exact test was chosen. These analyses were explorative.

Calculation of *p*-values was performed using SPSS (version 28.0, IBM Corp., Armonk, NY, USA). All *p*-values were used as descriptive measures. No adjustment for multiple testing was performed.

## Results

### Participants’ characteristics: general questions

In total, 70 physicians responded to the web-based survey by May 2023: 42 of them were radiation oncologists, 12 were medical oncologists, 14 head and neck surgeons, and 2 physicians covered several specialties. Of the respondents 48 (68.6%) worked at a low- or intermediate-volume center with up to 100 annually treated HNC patients. There was a relatively equal distribution of clinical experience among participants (*p* = 0.35). Most participants (*n* = 52, 74.3%) were affiliated with a university hospital. Participants’ characteristics are summarized in Table 1.

**Table 1:** Participants’ characteristics

	Participants *n* = 70 (100%)	*p*-value
**Section 1: general questions**		
*Age (years; mean* *±* *SD)*	38 ± 10.6	–
*Gender*		< 0.001^a^*
Female	39 (55.7%)	
Male	30 (42.9%)	
Diverse	1 (1.4%)	
*Clinical experience (years)*		0.35^a^
< 5	20 (28.6%)	
5–10	20 (28.6%)	
≤ 20	19 (27.1%)	
> 20	11 (15.7%)	
*Field of expertise*		< 0.001^a^*
Radiation oncology	42 (60%)	
Medical oncology	12 (17.1%)	
Otorhinolaryngology/head and neck surgery	14 (20%)	
Others (e.g., several areas)	2 (2.9%)	
*Number of treated HNC patients/year*		0.002^a^*
< 50	19 (27.1%)	
50–100	29 (41.4%)	
101–200	18 (25.7%)	
> 200	4 (5.7%)	
*Type of institution*		< 0.001^a^*
University hospital	52 (74.3%)	
Other hospital (nonuniversity, municipal)	9 (12.9%)	
Medical care center or private practice	9 (12.9%)	
**Section 2: current policies for tube feeding at the respective institution**		
*Indication for prophylactic gastric/nutritional feeding tube set within an interdisciplinary tumor board*		< 0.001^a^*
Yes	13 (18.6%)	
No	19 (27.1%)	
Occasionally/sometimes	32 (45.7%)	
Not attending a tumor board on a regular basis	6 (8.6%)	
*Use of an SOP governing the indication for feeding tube placement*		< 0.001^b^*
Yes	20 (28.6%)	
No	50 (71.4%)	
*Validated nutritional screening performed before planned CRT*		< 0.001^b^*
Yes	16 (22.8%)	
No	54 (77.1%)	
*Placement of prophylactic PEG tube is routinely performed before CRT*		< 0.001^b^*
Yes	56 (80%)	
No	14 (20%)	
*A patient undergoing chemoradiotherapy presents with dysphagia 4 weeks after treatment start. Swallowing ability could not have been improved (e.g., by means of speech therapy and/or pain management and intravenous fluid administration). You are considering inserting a feeding tube. What procedure does your institution follow?*		< 0.001^b^*
Insertion of a PEG tube	63 (90%)	
Insertion of a nasogastric tube/transnasal feeding tube	7 (10%)	
**Section 3: personal expert opinion**		
*The use of PEG tubes in daily clinical practice in HNC patients should …*		< 0.001^a^*
increase.	31 (44.3%)	
not change (neither increase nor decrease).	37 (52.9%)	
decrease.	2 (2.9%)	
*Have you seen any serious complications (including patient death) associated with PEG tube placement in your clinical career?*		0.01^b^*
Yes	46 (65.7%)	
No	24 (34.3%)	

*SD* standard deviation, *HNC* head and neck cancer, *SOP* standard operating procedure, *CRT* chemoradiotherapy, *PEG* percutaneous endoscopic gastrostomy

*Statistically significant *p*-value

^a^Chi-square test

^b^Test for binomial distribution

The comparison between radiation oncologists and other disciplines (medical oncologists and ENT) revealed that there were relatively more radiation oncologists treating up to 100 HNC/year (54.8%), while other disciplines were treating up to 200 (28.6%) or even more than 200 HNC patients (7.1%) per year (*p* = 0.03). Considering other general participant characteristics, we did not observe further differences (Table 2).

**Table 2:** Comparison of radiation oncologists’ responses with other specialties

	Radiation oncologists *n* = 42 (60%)	Other specialties *n* = 28 (40%)	*p*-value
**Section 1: general questions**			
*Age (years; mean* *±* *SD)*	37.3 ± 11.9	39.2 ± 8.5	0.43^a^
*Gender*			0.38^b^
Female	25 (59.5%)	14 (50%)	
Male	17 (40.5%)	13 (46.4%)	
Diverse	0	1 (3.6%)	
*Clinical experience (years)*			0.92^b^
< 5	13 (31%)	7 (25%)	
5–10	11 (26.2%)	9 (32.1%)	
10–20	11 (26.2%)	8 (28.6%)	
> 20	7 (16.7%)	4 (14.3%)	
*Number of treated HNC patients/year*			0.03^b^*
< 50	7 (16.7%)	12 (42.9%)	
50–100	23 (54.8%)	6 (21.4%)	
101–200	10 (23.8%)	8 (28.6%)	
> 200	2 (4.8%)	2 (7.1%)	
*Type of institution*			0.35^b^
University hospital	31 (73.8%)	21 (75%)	
Other hospital (nonuniversity, municipal)	4 (9.5%)	5 (17.9%)	
Medical care center or private practice	7 (16.7%)	2 (7.1%)	
**Section 2: current policies for tube feeding at the respective institution**			
*Indication for prophylactic gastric/nutritional feeding tube set within an interdisciplinary tumor board*			0.02^b^*
Yes	4 (9.5%)	9 (32.1%)	
No	12 (28.6%)	7 (25%)	
Occasionally/sometimes	24 (57.1%)	8 (28.6%)	
Not attending a tumor board on a regular base	2 (4.8%)	4 (14.3%)	
*Use of an SOP governing the indication for feeding tube placement*			0.01^c^*
Yes	7 (16.7%)	13 (46.4%)	
No	35 (83.3%)	15 (53.6%)	
*Validated nutritional screening performed before planned CRT*			0.54^c^
Yes	35 (83.3%)	21 (75%)	
No	7 (16.7%)	7 (25%)	
*Placement of prophylactic PEG tube is routinely performed before CRT*			0.54^c^
Yes	35 (83.3%)	21 (75%)	
No	7 (16.7%)	7 (25%)	
*A patient undergoing chemoradiotherapy presents with dysphagia 4 weeks after treatment start. Swallowing ability could not have been improved (e.g., by means of speech therapy and/or pain management and intravenous fluid administration). You are considering inserting a feeding tube. What procedure does your institution follow?*			0.11^c^
Insertion of a PEG tube	40 (95.2%)	23 (82.1%)	
Insertion of a nasogastric tube/transnasal feeding tube	2 (4.8%)	5 (17.9%)	
**Section 3: personal expert opinion**			
*The use of PEG tubes in daily clinical practice in HNC patients should …*			0.03^b^*
increase.	23 (54.8%)	8 (28.6%)	
not change (neither increase nor decrease).	17 (40.5%)	20 (71.4%)	
decrease.	2 (4.8%)	0	
*Have you seen any serious complications (including patient death) associated with PEG tube placement in your clinical career?*			0.04^c^*
Yes	32 (76.2%)	14 (50%)	
No	10 (23.8%)	14 (50%)	

*SD* standard deviation, *HNC* head and neck cancer, *SOP* standard operating procedure, *CRT* chemoradiotherapy, *PEG* percutaneous endoscopic gastrostomy

*Statistically significant *p*-value

^a^Mann-Whitney U test

^b^Chi-square test

^c^Fisher’s exact test

### Participants’ characteristics: tube feeding policies in chemoradiotherapy

The majority of participants (77.1%, *n* = 54) did not perform standard nutritional screening at their institution prior to planned CRT. The indication for PEG tube placement prior to CRT was made in 45.7% (*n* = 32) within an interdisciplinary tumor board, while 27.4% (*n* = 19) do not discuss this topic. Moreover, most respondents were in favor of prophylactic PEG tube placement in case of planned CRT, while 20% (*n* = 14) decided against prophylactic placement (Table 1). In most cases, no SOP was used for the indication of a feeding tube. When asked on a scale of 0 to 10 (with 0 = absolutely useless to 10 = extremely useful) how useful it would be to introduce an SOP at the respective institution (if one does not yet exist), one (1.4%) participant answered with 0–3, 15 (21.4%) participants with 4–6, and 54 (77.1%) participants with 7–10. Regarding the responsibility for PEG tube placement, most participants answered that gastroenterology/endoscopy, followed by interventional radiology, followed by general surgery, was in charge (multiple answers possible).

The comparison between radiation oncologists and the other two surveyed disciplines (medical oncologists and ENT/otolaryngologists) revealed differences in the determination/indication setting of feeding tube placement within a multidisciplinary tumor board (*p* = 0.02) and the use of an SOP (standard operating procedure; *p* = 0.01). Accordingly, among medical oncologists and otolaryngologists, approximately half of the respondents each affirmed or denied the use of an SOP for the indication of feeding tube placement, while the proportion not using an SOP at their institution predominated among radiation oncologists (35/42 radiation oncologists; 83.3%; *p* = 0.01; Table 2).

### Participants’ characteristics: participants’ expert opinion(s)

Overall, only a minority of respondents thought that the use of PEG tubes should decrease in clinical practice (*n* = 2; 2.9%; *p* < 0.001), while the majority indicated that use of feeding tubes should remain as it is (neither increase nor decrease; *n* = 37; 52.9%; *p* < 0.001). A similar trend seemed to emerge when comparing radiation oncologists and the other two disciplines surveyed. However, most radiation oncologists even stated that the use of PEG tubes should increase in clinical practice (*n* = 23; 54.8%; *p* = 0.03). At the same time, most participants (*n* = 46; 65.7%) have seen serious complications associated with PEG tube placement (*p* = 0.01). This proportion is even higher among radiotherapy colleagues (*n* = 32; 76.2%) compared to the other two disciplines (*n* = 14; 50%; *p* = 0.04).

Participants were asked to rate on a scale of 0–10 (0 = absolutely untrue and 10 = absolutely true) whether emergency hospitalization under initially started outpatient chemoradiotherapy occurs more frequently in patients without a prophylactically placed PEG tube. Eight participants (11.4%) answered with 0–3, 19 (27.1%) participants with 4–6, and 43 (61.4%) participants with 7–10.

**Fig. 1:**
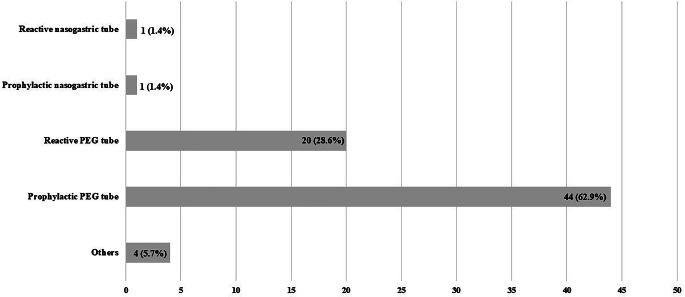
Participants’ answers on case report no. 1. *PEG* percutaneous endoscopic gastrostomy

**Fig. 2:**
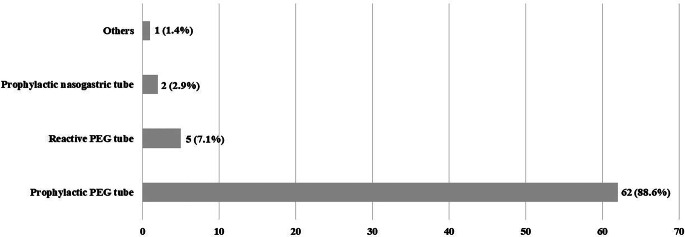
Participants’ answers on case report no. 2. *PEG* percutaneous endoscopic gastrostomy

**Fig. 3:**
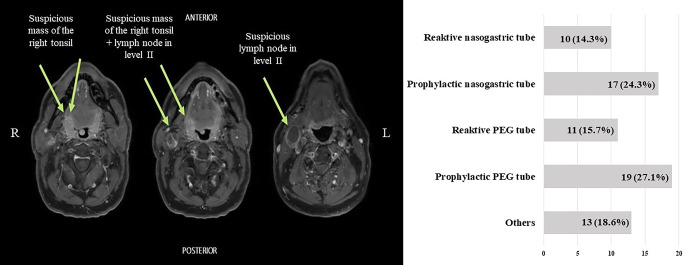
Participants’ answers on case report no. 3. The x‑axis represents the number of responses given. *PEG* percutaneous endoscopic gastrostomy, *MRI* magnetic resonance imaging, *BMI* body mass index, *ENT* ear, nose, throat

In addition, participants were confronted with three case reports and asked for their expert opinion. Their answers are shown in Figs. 1, 2, and 3.

## Discussion

Our nationwide web-based survey investigated current policies and practices of tube feeding in HNC patients among different treatment centers in Germany. It provides valuable insights into the characteristics and opinions of healthcare professionals, specifically radiation oncologists, medical oncologists, and ENT surgeons/otolaryngologists. More than 70% of our respondents stated that they neither conduct routine nutritional screening nor that they rely on SOPs concerning EN management. However, over 80% of the respondents support the insertion of prophylactic PEG tubes before planned CRT.

Our study included 70 physicians from various specialties involved in HNC care. Most participants worked at low- and intermediate-volume centers (with fewer than 50 or up to 100 HNC cases per year, respectively), and were mostly affiliated with university hospitals. This distribution reflects the diverse backgrounds of healthcare providers involved in the management of HNC patients and also underlines the structure of the German healthcare system: in addition to university clinics or hospitals that are certified cancer centers alone or in a network, outpatient treatment can also take place in specialist private practices. However, data have shown that the treatment of HNC patients at high-volume centers was associated with a survival benefit [22, 23]. Although the definition of a high-volume center varies across different publications, these data apply to surgical as well as to conservative (including radiotherapy) disciplines, emphasizing that interdisciplinary experience matters [24]. However, the importance of nutritional medical support or nutritional counselling services is not addressed in these analyses. The uneven distribution in terms of our participants’ patient volume (or cases/year) underlines the need for standardized protocols not only in oncologic treatment decision-making but also in the management of nutritional complications.

Our study revealed that a significant proportion of participants do not perform standardized nutritional screening before planned CRT. There are several studies that confirm the benefits of pretherapeutic nutritional screening [25, 26]. According to our experience, this non-performance of a pretherapeutic nutritional screening is not attributable to a general physicians’ rejection, but rather to workload compression and staff shortage in the German healthcare system.

Since dysphagia may lead to malnutrition, which, in turn, may negatively impact overall survival of head and neck cancer patients [27], radiation-induced dysphagia remains a focus of ongoing scientific research. Accordingly, there are not only several studies on its prevention [7, 8], but also on the correct timing of PEG tube insertion. The Swall PEG study is particularly noteworthy here [12]: as a randomized, controlled phase III study, its primary endpoint investigated patient-reported outcomes related to swallowing and patients’ health-related quality of life. The authors have announced their intention to publish their results in a peer-reviewed journal [12]. Other efforts address the avoidance of radiation-associated dysphagia by means of optimized radiation planning (dysphagia-optimized intensity-modulated radiotherapy) with dose constraints to the superior and middle pharyngeal constrictor muscle or inferior pharyngeal constrictor muscle up to a mean dose of 50 Gy. The results imply that this dose-sparing of the swallowing muscles leads to an improvement in patient-reported swallowing [7].

There is a preference for prophylactic PEG tube placement among most our respondents, which aligns with the notion that early placement may reduce the risk of malnutrition and hospital readmissions [28]. Yanni et al. performed a retrospective analysis of 152 patients undergoing surgery, radiotherapy, or chemotherapy due to HNC. These authors concluded that PEG tubes were advantageous regarding hospital readmissions, relative weight loss at 6 weeks, dysphagia, severe malnutrition, and the patients’ health status [28]. On the contrary, Madhoun et al. surveyed HNC patients between 2004 and 2006. They reported a high number of unnecessary PEG tube placements when feeding tube insertion was performed prophylactically. Thus, only 47.8% of their patients with prophylactic PEG tubes either did not use their feeding tube at all or used it less than two weeks [29]. Overall, the correct timing of PEG tube placement remains a controversial point of discussion that should be weighed individually depending on the existing nutritional risk factors. Of course, this presupposes that the risk factors are known or have been determined within the framework of an individual nutritional risk screening [26].

Interestingly, we noticed differences between radiation oncologists and other disciplines regarding the indication setting and the use of SOPs. The proportion of radiation oncologists using an SOP appeared to be lower compared to other specialties. Although this finding could be biased by the imbalance between the professional groups, possible reasons for this could lie in the dynamic field of radiation oncology: Rapid changes in treatment practices may outpace the development or updating of SOPs, leading radiation oncologists to rely more on evolving evidence and expert consensus rather than fixed SOPs. Therefore, the unique and individualized nature of radiation therapy, interdisciplinary collaboration dynamics, the evolving nature of treatment practices, and the absence of universally accepted guidelines in certain contexts may lead to a lower use of SOPs regarding the indication for enteral feeding tubes.

SOPs aim at identifying potential risks and outlining preventive measures. Risk management is recommended to ensure safety and quality in radiation oncology [30, 31]. Bearing in mind that available data are still limited on the topic of risk management in German radiation oncology, published data on the handling of enteral or parenteral feeding due to radiation-induced dysphagia are also very sparse. Although some of our respondents follow internal institute SOPs on this topic, the majority of our interviewed radiation oncologists do not use SOPs, thus implying a potential need for standardized guidelines to warrant consistent and evidence-based decision-making regarding radiation-induced swallowing complications.

Our respondents’ opinions on the use of PEG tubes varied, with the majority indicating that the use of feeding tubes should remain unchanged. Notably, radiation oncologists favored an increasing use of PEG tubes, possibly driven by concerns about treatment interruptions due to severe treatment-related toxicities. This viewpoint has been reflected by some studies suggesting that prophylactic PEG tube placement may reduce treatment interruptions [32] and improve outcomes [33].

Overall, the abovementioned perspectives reflect the ongoing debate in the literature regarding the appropriate use and timing of PEG tubes [29, 34]. A significant proportion of our participants reported severe complications associated with PEG tube placement. This underscores the necessity for careful consideration of risks and benefits before PEG tube insertion. Severe complications including infection and dislodgment as well as tube-related morbidity and (in rare cases) even mortality have been reported previously [14]. For decision-making, the EHNS-ESMO-ESTRO (European Head and Neck Society - European Society of Medical Oncology - European Society for Radiotherapy and Oncology) clinical practice guideline can be a source of support: in case of present malnutrition (defined by a weight loss of more than 10% over 6 months before diagnosis), an enteral feeding tube is recommended. When long-term feeding tube dependence is foreseeable, PEG tubes should be preferred over nasogastric tubes [35]. Moreover, there are some publications that at least provide information on other factors relevant for development of dysphagia and, thus, dependence on a feeding tube during ongoing therapy. These factors include the radiation dose to the swallowing muscles or the radiation volume [11, 36, 37] and the tumor localization [38], as well as weight loss at diagnosis [11] and age [11]. Although validated nutritional screening tools focusing on weight loss exist [9], there are no validated universal risk scores that combine radio-oncologic risks (e.g., radiation dose to the swallowing muscles) and clinical/nutritional risks (e.g., weight loss in the past 6 months).

The different perspectives are also reflected in the patient cases described: in question no. 13, our respondents were asked to decide whether they would rather have a PEG or nasogastric tube inserted if their patient presented with (otherwise uncontrollable) dysphagia 4 weeks after starting chemoradiotherapy. The same question about the chosen method of reactive tube feeding was asked in the Scandinavian survey by Ilmarinen et al. [17]. Their survey included otorhinolaryngology head and neck (ORL-HN) surgeons and oncologists (including medical oncologists and radiation oncologists). Most of their participants (> 65%) would have chosen a nasogastric tube over a PEG tube. Interestingly, 63% of our participants would have opted to have a PEG tube inserted. The tendency to use a PEG tube in this situation also prevailed in the interprofessional comparison in our survey. This discrepancy between the two studies may be due to the chosen subject collective. While in the survey by Ilmarinen et al., no explicit distinction was made between radiation oncologists and medical oncologists, radiation oncologists dominate in our survey. It is well known that side effects after chemoradiotherapy often persist for several weeks after the end of treatment. Therefore, we assume that this was the reason why our respondents decided to have a PEG tube inserted.

In question no. 16, our participants were asked which tube-feeding strategy they would choose in a 55-year-old male patient suffering from p16-positive squamous cell carcinoma of the base of the tongue (cT2 cN1 cM0) with a BMI of 25 kg/m^2^ and without significant weight loss, history of pain, or swallowing problems, when chemoradiotherapy is planned [17] (55% percent of our Scandinavian colleagues preferred a reactive nasogastric tube [17]). For a 65-year-old male patient with hypopharyngeal carcinoma (cT2 cN2b cM0) and swallowing difficulties, 67% of the Scandinavian respondents opted for a prophylactic PEG tube. For both situations, no differences were observed between head and neck surgeons and oncologists by Ilmarinen et al. [17]. In both cases, German physicians mostly preferred a prophylactic PEG tube (62.9 and 88.6%, respectively).

The third and last patient case described the case of a 59-year-old patient with right-sided oropharyngeal carcinoma (good general health, BMI of 23 kg/m^2^), who opted for a transoral resection with neck dissection. Participants were asked how to proceed regarding tube feeding. There was no clear trend regarding one option. We were surprised that a prophylactic PEG tube was also considered in 27.1%.

Although our survey on PEG tube placement in head and neck cancer patients undergoing CRT provided valuable insights, there are limitations to be acknowledged. Firstly, the study’s sample size, consisting of 70 respondents, may not fully represent the diverse range of German healthcare professionals involved in HNC treatment. Therefore, our findings may not be generalizable to all practice settings. Our survey is based on participant opinions and their practices, which does not guarantee guideline adherence. Accordingly, we were dealing with self-reported data and cannot exclude a recall bias. An important limitation is the disbalance between the professional groups: since radiation oncologists predominate in this case, conclusions drawn from the SUFFEETUBE project cannot be generalized automatically to other medical specialties. At the same time, we were able to show that an unequal approach to feeding tubes exists throughout Germany (irrespective of the professional group). This insight may provide an impetus for standardization and the development of guidelines. At the same time, it must also be emphasized that not all clinically active ENT physicians, oncologists, and radiation oncologists throughout Germany were contacted. Therefore, a selection bias cannot be ruled out.

## Conclusion

In conclusion, the policies and methods of tube feeding vary considerably between the individual institutions and specialties in Germany. However, uniform protocols are difficult to establish when an individualized approach is to be maintained. Still, a baseline nutritional risk screening (e.g., NRS2002 or MUST) could simplify decision-making in clinical practice. Further improvements should aim at the development of a modified risk screening including nutritional, clinical, and dosimetric factors to decide upon the necessity of prophylactic or reactive nutritional measurements.
